# Nuclear ELAC2 overexpression is associated with increased hazard for relapse after radical prostatectomy

**DOI:** 10.18632/oncotarget.27132

**Published:** 2019-08-13

**Authors:** Cornelia Schroeder, Elham Navid-Hill, Jan Meiners, Claudia Hube-Magg, Martina Kluth, Georgia Makrypidi-Fraune, Ronald Simon, Franziska Büscheck, Andreas M. Luebke, Cosima Goebel, Dagmar S. Lang, Sören Weidemann, Emily Neubauer, Andrea Hinsch, Frank Jacobsen, Patrick Lebok, Uwe Michl, Dirk Pehrke, Hartwig Huland, Markus Graefen, Thorsten Schlomm, Guido Sauter, Doris Höflmayer

**Affiliations:** ^1^ Institute of Pathology, University Medical Center Hamburg-Eppendorf, Hamburg, Germany; ^2^ General, Visceral and Thoracic Surgery Department and Clinic, University Medical Center Hamburg-Eppendorf, Hamburg, Germany; ^3^ Martini-Clinic, Prostate Cancer Center, University Medical Center Hamburg-Eppendorf, Hamburg, Germany; ^4^ Department of Urology, Charité - Universitätsmedizin Berlin, Berlin, Germany

**Keywords:** ELAC2, HPC2, prostate cancer, prognosis, tissue microarray

## Abstract

ELAC2 is a ubiquitously expressed enzyme potentially involved in tRNA processing and cell signaling pathways. Mutations of the ELAC2 gene have been found to confer increased prostate cancer susceptibility in families. ELAC2 protein expression was analyzed by immunohistochemistry in 9,262 patients and Kaplan-Meier curves of PSA recurrence-free survival were calculated in 8,513 patients treated with radical prostatectomy. Nuclear ELAC2 staining was observed in 60.8% of prostate cancers. It was weak in 26.3%, moderate in 26.6% and strong in 7.9%. Strong nuclear ELAC2 expression was associated with advanced tumor stage, nodal metastasis, higher Gleason grade, presence of *TMPRSS2*:ERG fusion, higher Ki67-labeling index and *PTEN* deletion. The difference in 1-, 5- and 10-year recurrence-free survival between strong and weak nuclear ELAC2 intensity is 7.2/13.8/17.6% in all cancers, 7.4/16.1/26.5% in the ERG negative subset, and 3.1/5.7/9.8% in the ERG positive subset. Regarding the univariate hazard ratio, PSA recurrence-free survival after prostatectomy for strong nuclear ELAC2 expression is 1.89 (1.64–2.10, *p* < 0.0001). It is independent of preoperative PSA-level, Gleason grade, pathological stage, surgical margin stage, and lymph node stage (multivariate hazard ratio 1.29 (1.11–1.49, *p* = 0.001). We conclude that nuclear ELAC2 expression is an independent prognostic marker for PSA recurrence-free survival after radical prostatectomy with a weak to moderate increase of the hazard ratio for biochemical relapse.

## INTRODUCTION

In Western societies, prostate cancer is the most prevalent cancer in males [1]. Most of these are indolent and only about 10% are highly aggressive. The established prognostic factors (Gleason grade, tumor extent in biopsies, preoperative prostate-specific antigen (PSA) level and clinical stage) are statistically powerful but not specific and sensitive enough to predict aggressive behavior for efficient individual treatment decisions.

The ELAC2 gene (alias RNase Z2, Hereditary Prostate Cancer locus 2 (HPC2)), located at chromosome 17p12, encodes a zinc phosphodiesterase. Its cellular role is poorly understood. Available data point towards multiple functions including transfer RNA processing [2–4], interaction with g-tubulin [5], and modification of transforming growth factor-ß (TGF-ß) pathway activity [6]. ELAC2 expression has been reported from a wide variety of normal tissues, supporting a role of the protein in many cell types [7]. ELAC2 is of particular interest in prostate cancer because sequence variants of this gene have been suggested to play a role in genetic susceptibility to hereditary and sporadic forms of the disease [8–13]. However, studies assessing the expression profile and putative prognostic role of the ELAC2 protein in prostate cancer are lacking.

Here we used a large and highly annotated tissue microarray (TMA) to study ELAC2 expression by immunohistochemistry.

## RESULTS

**Figure 1:**
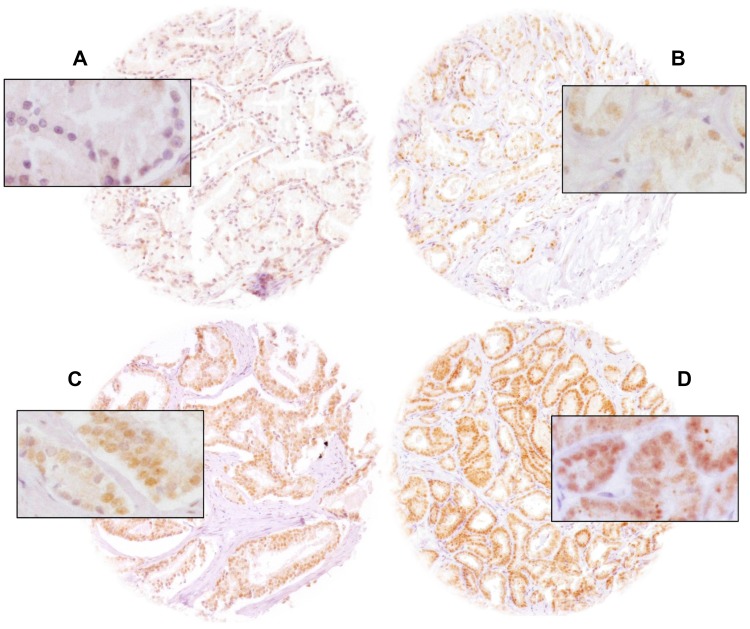
Representative pictures of prostate cancer with (**A**) negative, (**B**) weak, (**C**) moderate and (**D**) strong nuclear ELAC2 staining. Magnification was 100×, insets 400× and spot size 600 μm.

In our TMA analysis, a total of 74.5% (9,262/12,427) of tumor samples were interpretable. Non-informative cases (25.5%, 3,165/12,427) either had no tissue at all or no cancer tissue in the TMA spot. ELAC2 staining was predominantly localized in the nucleus of invasive prostate cancer cells. Nuclear ELAC2 staining was seen in 5,634 of 9,262 (60.8%) prostate cancers and was considered weak in 26.3%, moderate in 26.6% and strong in 7.9%. Representative images of nuclear ELAC2 staining are given in Figure 1. In order to find differences between normal and cancer, tissue spots containing both normal and cancer glands were evaluated. It showed that ELAC2 staining in cancer glands was typically stronger as compared to adjacent normal prostatic glands (Supplementary Figure 1).

### Association with *TMPRSS2:ERG* fusion status and ERG protein expression

**Figure 2:**
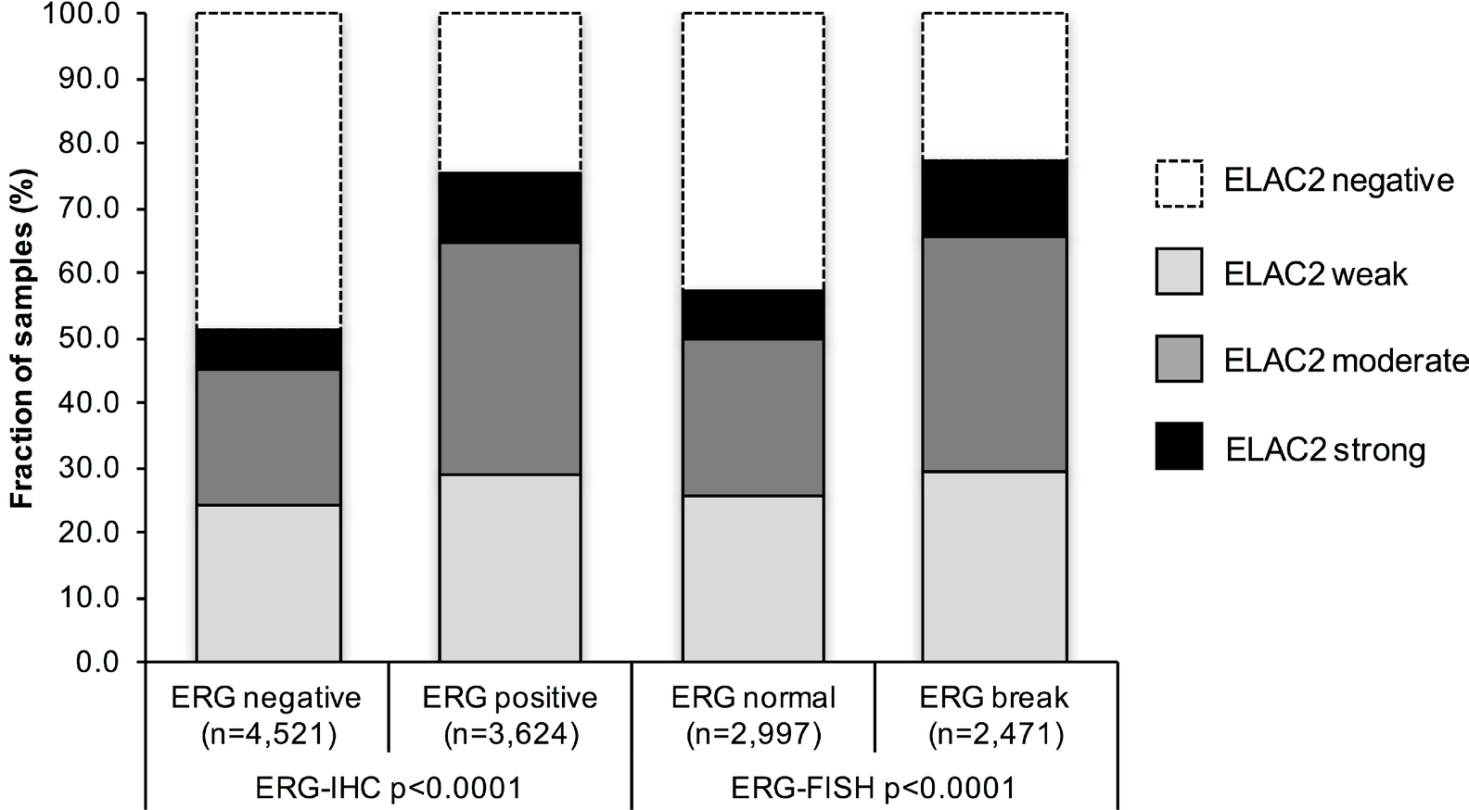
Association between nuclear ELAC2 staining and ERG status determined by immunohistochemistry (IHC) and fluorescence *in situ* hybridization (FISH).

*TMPRSS2:ERG* fusion status obtained by FISH was available from 5,468 patients, by IHC from 8,145 patients and by both ERG FISH and IHC from 5,268. Matching results (ERG IHC positive and break by FISH) were found in 5,041 of 5,268 (92.2%) cancers. High-level nuclear ELAC2 staining was associated with *TMPRSS2:ERG* rearrangement and ERG expression in prostate cancers (*p* < 0.0001 each; Figure 2). For example, moderate or strong nuclear ELAC2 staining was seen in 48.1% of cancers with *TMPRSS2:ERG* fusion detected by FISH but in only 31.6% of cancers without such rearrangement (*p* < 0.0001).

### Associations with tumor phenotype

Increased nuclear ELAC2 expression was linked to high Gleason grade (*p* < 0.0001), higher pathological tumor stage (*p* < 0.0001) and positive nodal status (*p* = 0.0003; Table 1). Subset analyses of ERG positive and ERG negative cancers revealed that these associations were stronger in ERG negative cancers (*p* < 0.0001 each, Table 2). In ERG positive cancers only high Gleason grade (*p* < 0.0001) remained as significantly linked to ELAC2 expression (Table 3).

**Table 1:** Association between ELAC2 staining and prostate cancer phenotype

Parameter	Evaluable	ELAC2 nuclear staining (%)				*P* value
	(*n*)	Negative	Weak	Moderate	Strong	
**Total**	9 262	39.2	26.3	26.6	7.9	
**Tumor stage**						
pT2	5 956	42.2	26.0	25.2	6.6	<0.0001
pT3a	2 088	34.8	27.4	28.5	9.3	
pT3b	1 130	31.2	26.4	30.4	12.0	
pT4	50	38.0	16.0	30.0	16.0	
**Gleason grade**						
≤3+3	2 120	52.5	23.4	20.0	4.1	<0.0001
3+4	4 953	37.2	26.8	28.0	7.9	
3+4 Tert.5	321	30.2	28.7	30.8	10.3	
4+3	944	33.6	28.1	28.0	10.4	
4+3 Tert.5	478	24.7	27.0	35.1	13.2	
≥4+4	440	30.7	27.7	28.2	13.4	
**Lymph node metastasis**						
N0	5 236	35.9	25.9	28.7	9.5	0.0003
N+	532	27.3	29.1	30.6	13.0	
**Preoperative PSA level (ng/ml)**						
<4	1 136	36.4	27.5	27.0	9.1	0.0624
4–10	5 508	39.6	25.9	26.9	7.6	
10–20	1 849	38.9	26.8	26.9	7.4	
>20	662	41.1	25.5	23.0	10.4	
**Surgical margin**						
Negative	7 325	39.7	26.1	26.4	7.8	0.2622
Positive	1 767	37.2	26.9	27.6	8.3	

**Table 2:** Association between ELAC2 staining and prostate cancer phenotype in the TMPRSS2: ERG fusion negative subset

Parameter	Evaluable	ELAC2 nuclear staining (%)				*P* value
	(*n*)	Negative	Weak	Moderate	Strong	
**Total**	4 521	48.7	24.0	21.0	6.2	
**Tumor stage**						
pT2	3 027	52.2	23.4	19.7	4.7	<0.0001
pT3a	914	46.3	25.4	21.2	7.1	
pT3b	543	33.1	25.8	28.2	12.9	
pT4	23	47.8	13.0	30.4	8.7	
**Gleason grade**						
≤3+3	959	65.8	19.7	12.6	1.9	<0.0001
3+4	2 359	48.2	24.8	21.4	5.6	
3+4 Tert.5	189	36.5	27.0	26.5	10.1	
4+3	490	40.6	27.3	23.3	8.8	
4+3 Tert.5	261	29.9	24.5	32.2	13.4	
≥4+4	260	33.5	23.8	30.0	12.7	
**Lymph node metastasis**						
N0	2 613	45.5	25.0	22.2	7.3	<0.0001
N+	255	31.4	23.9	31.4	13.3	
**Preoperative PSA level (ng/ml)**						
<4	461	46.0	24.5	20.8	8.7	0.0053
4–10	2 663	49.6	23.8	21.2	5.4	
10–20	992	46.8	25.2	22.2	5.8	
>20	366	50.0	21.9	17.5	10.7	
**Surgical margin**						
Negative	3 586	49.3	23.8	20.8	6.1	0.4911
Positive	855	46.8	23.9	22.7	6.7	

**Table 3:** Association between ELAC2 staining and prostate cancer phenotype in the TMPRSS2: ERG fusion positive subset

Parameter	Evaluable	ELAC2 nuclear staining (%)				*P* value
	(*n*)	Negative	Weak	Moderate	Strong	
**Total**	3 624	24.6	28.9	35.6	10.8	
**Tumor stage**						
pT2	2 130	25.0	29.2	35.4	10.4	0.2855
pT3a	981	22.6	29.7	36.4	11.3	
pT3b	475	27.2	27.2	34.5	11.2	
pT4	21	23.8	14.3	33.3	28.6	
**Gleason grade**						
≤3+3	770	33.9	27.7	30.9	7.5	<0.0001
3+4	2 064	22.4	29.1	37.4	11.1	
3+4 Tert.5	110	17.3	30.9	39.1	12.7	
4+3	358	23.7	28.2	35.2	12.8	
4+3 Tert.5	185	17.3	29.7	39.5	13.5	
≥4+4	135	24.4	32.6	29.6	13.3	
**Lymph node metastasis**						
N0	2 085	22.0	27.1	37.9	12.9	0.1541
N+	232	22.4	33.6	31.9	12.1	
**Preoperative PSA level (ng/ml)**						
<4	496	24.4	29.0	35.5	11.1	0.9981
4–10	2 184	24.4	28.8	35.9	11.0	
10–20	660	25.2	28.8	35.9	10.2	
>20	234	26.1	30.3	33.3	10.3	
**Surgical margin**						
Negative	2 818	24.8	28.7	35.5	11.0	0.8555
Positive	738	24.3	30.1	35.5	10.2	

### Associations with other key genomic deletions

**Figure 3:**
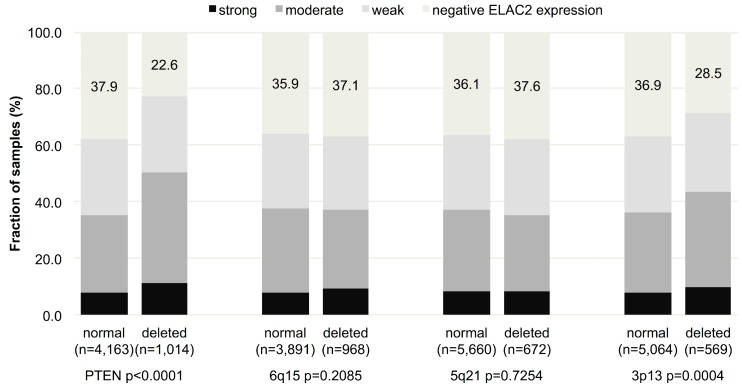
Association between nuclear ELAC2 staining and 10q23 (*PTEN),* 5q21 (*CHD1*), 6q15 (*MAP3K7*), and 3p13 (*FOXP1*) – deletion. Numbers indicate the percentage of ELAC2-negative cancers in each group.

To learn whether ELAC2 expression might be particularly linked to recurrent genomic deletions in prostate cancer, nuclear ELAC2 expression was compared to 10q23 (*PTEN*), 3p13 (*FOXP1*), 6q15 (*MAP3K7*) and 5q21 (*CHD1*) deletion. Increased nuclear ELAC2 expression was significantly associated with *PTEN* deletion (*p* < 0.0001) and 3p13 deletion (*p* = 0.0004), although the differences in absolute numbers were only small. For example, the fraction of ELAC2-negative cancers decreased from 37.9% in cancers with normal PTEN copy numbers to 22.6% in cancers with PTEN deletion (Figure 3).

### Tumor cell proliferation

**Figure 4:**
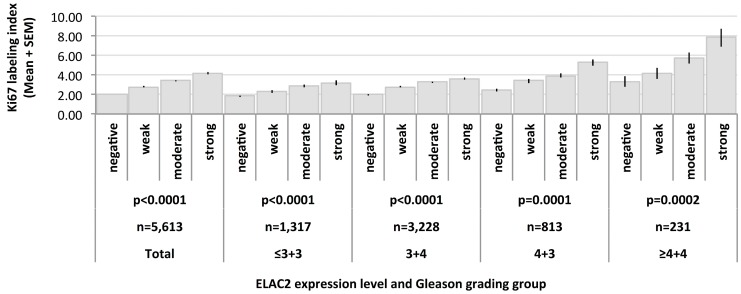
Association between Ki67 labeling index and ELAC2 expression level in various Gleason grading groups.

High level nuclear ELAC2 staining was associated with increased Ki67 labeling index (Ki67LI) (Figure 4). The average Ki67LI increased from 2.02 in cancers lacking nuclear ELAC2 expression to 4.18 in cancers with high ELAC2 levels (*p* < 0.0001). Subset analysis of of cancers with identical Gleason grade (≤3+3: *p* < 0.0001, 3+4: *p* < 0.0001, 4+3: *p* < 0.0001, ≥4+4: *p* = 0.0002; Figure 4) revealed that this association was independent from the Gleason grade.

### Associations with PSA recurrence

**Figure 5:**
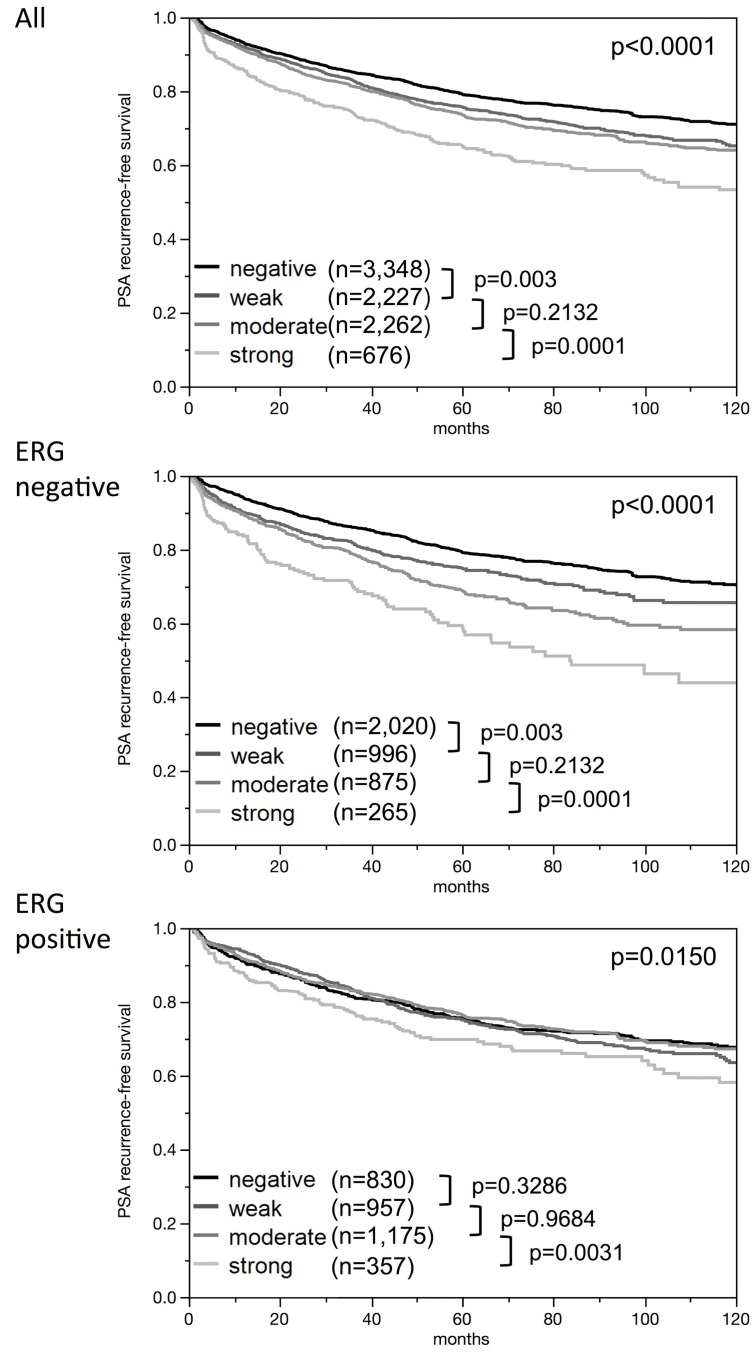
Kaplan-Meier plots of prostate specific antigen (PSA) recurrence after radical prostatectomy and ELAC2 staining in all cancers, the ERG negative subset, and the ERG positive subset. *P*-values [log-rank] are uncorrected.

**Figure 6:**
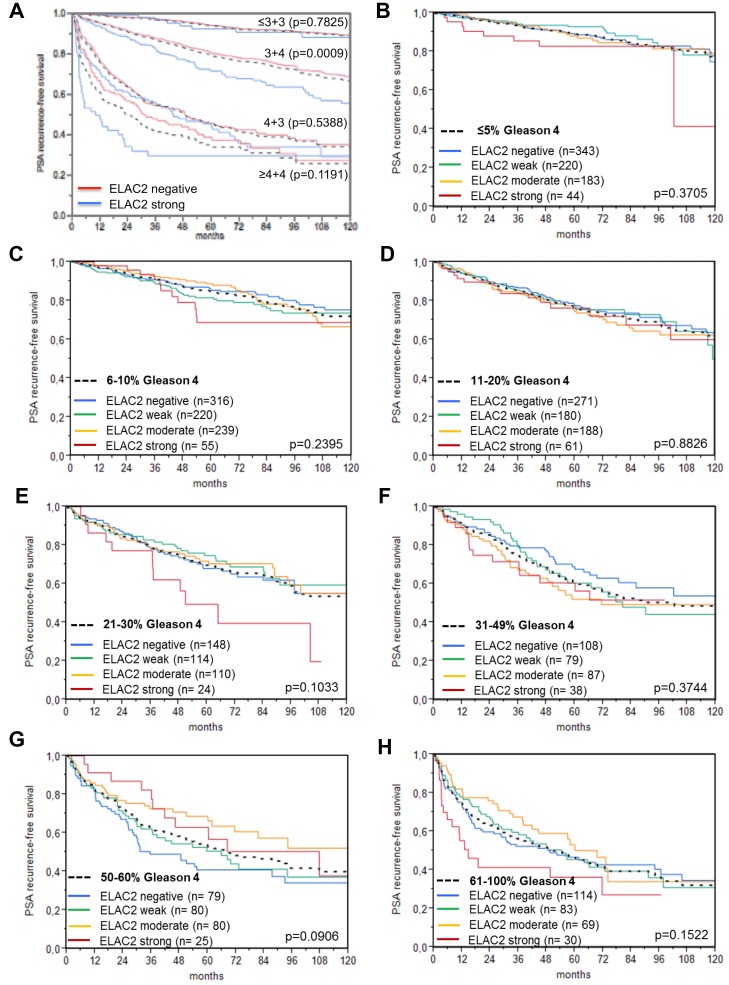
Prognostic impact of negative, (weak, moderate) and strong ELAC2 expression level in subsets of cancers defined by (**A**) the classical Gleason score (black dotted lines) and (**B**–**H**) the quantitative Gleason score categories (black dotted lines) defined by the percentage of Gleason 4 patterns: (**B**) ≤5%, (**C**) 6–10%, (**D**) 11–20%, (**E**) 21–30%, (**F**) 31–49%, (**G**) 50–60%, and (**H**) 61–100% Gleason 4 pattern. *P*-values [log-rank] are uncorrected.

High-level nuclear ELAC2 expression was linked to earlier biochemical recurrence (Figure 5). The in 1-, 5- and 10-year recurrence-free survival differed between strong and weak nuclear ELAC2 intensity levels by 7.2 /13.8/17.6% in all cancers, 7.4/16.1/26.5% in the ERG negative subset and 3.1/5.7/9.8% in the ERG positive subset. Furthermore, we did subset analyses in cancers with identical classical and quantitative Gleason scores (Figure 6). Here, nuclear ELAC2 staining provided clear-cut prognostic information beyond the classical Gleason score in the Gleason group 3+4 (*p* = 0.0009, Figure 6A). This effect was lost by further subgrouping into quantitative Gleason categories (Figure 6B–6H).

### Multivariate analysis

Cox regression analysis was used to calculate the hazard ratios for PSA recurrence-free survival of negative, weak, moderate, and strong nuclear ELAC2-intensitiy levels. In the univariable model there was a moderate effect, with a hazard ratio between strong versus negative nuclear ELAC2 expression of 1.89 (95%CI 1.64–2.10). In the 4 multivariable models, nuclear ELAC2 expression provided independent prognostic information in all scenarios (Table 4). The hazard ratios were slightly higher in the ERG negative subset when compared with the ERG positive subset (Table 5).

**Table 4:** Hazard ratios (95% confidence intervals) for biochemical relapse after prostatectomy for established risk factors and nuclear ELAC2 expression in various scenarios

Model		Scenario 1	Scenario 2	Scenario 3	Scenario 4
Variable	Category (*N*)	8 232	8 362	8 485	8 473
Gleason grade biopsy	≥4+4 vs. 4+3 vs. 3+4 vs. ≤3+3	4.14 (3.63–4.69)^***^			
cT stage	T2c vs. T1c	2.23 (1.73–2.71)^***^	2.05 (1.63–2.53)^***^		
Preoperative PSA level	≥20 vs. 11–20 vs. 4–10 vs.<4	3.88 (3.19–4.74)^***^	2.98 (2.46–3.64)^***^	2.08 (1.71–2.53)^***^	1.91 (1.57–2.33)^***^
Nuclear ELAC2 expression	Strong vs. mod. vs. weak vs. neg.	1.58 (1.35–1.83)^***^	1.42 (1.22–1.64)^***^	1.33 (1.15–1.54)^**^	1.29 (1.11–1.49)^**^
Gleason grade prostatectomy	≥4+4 vs. 4+3 vs. 3+4 vs. ≤3+3		13.3 (10.9–16.1)^***^	6.48 (5.27–7.97)^***^	5.40 (4.36–6.70)^***^
pT stage	T4 vs. T3 vs. T2			3.14 (2.77–3.55)^***^	2.83 (2.48–3.22)^***^
Surgical margin status	R1 vs. R0			1.40 (1.27–1.54)^***^	1.40 (1.27–1.54)^***^
Nodal stage	N+ vs. N0				1.42 (1.24–1.63)^***^

Scenario 1 combines preoperatively available parameter (preoperative Gleason grade obtained on the original biopsy, clinical tumor (cT) stage, and preoperative PSA) with the postoperative ELAC2 expression at the negative, low (weak and moderate) and strong intensity levels. In scenario 2 the biopsy Gleason is replaced by the Gleason grade obtained on radical prostatectomy (RPE). In scenario 3, cT-stage is superseded by pathological tumor (pT) stage and surgical margin (R) status. In scenario 4 the lymph node (pN) stage is added. Asterisk indicate significance level: ^*^ *p* ≤ 0.05, ^**^ *p* ≤ 0.001, and ^***^ *p* ≤ 0.0001.

**Table 5:** Hazard ratios (95% confidence intervals) for biochemical relapse after prostatectomy for established risk factors and nuclear ELAC2 expression in the ERG negative and positive subset

Model		Scenario 1		Scenario 4	
ERG subset		Positive	Negative	Positive	Negative
Variable	Category (*N*)	3 291	4 033	3 301	4 141
Gleason grade biopsy	≥4+4 vs. 4+3 vs. 3+4 vs. ≤3+3	5.05 (4.08–6.23)^***^	3.51 (2.94–4.19)^***^		
cT stage	T2c vs. T1c	2.23 (1.62–3.00)^***^	2.03 1.36–2.91)^**^		
Preoperative PSA level	≥20 vs. 11–20 vs. 4–10 vs.<4	4.22 (3.11–5.77)^***^	3.17 (2.39–4.25)^***^	1.87 (1.38–2.55)^***^	1.70 (1.29–2.28)^**^
Nuclear ELAC2 expression	Strong vs. mod. vs. weak vs. neg.	1.42 (1.13–1.79)^*^	1.67 (1.33–2.08)^***^	1.18 (0.94–1.48)	1.43 (1.14–1.77)^*^
Gleason grade prostatectomy	≥4+4 vs. 4+3 vs. 3+4 vs. ≤3+3			7.56 (5.27–10.9)^***^	4.84 (3.57–6.58)^***^
pT stage	T4 vs. T3 vs. T2			3.03 (2.45–3.73)^***^	2.66 (2.21–3.20)^***^
Surgical margin status	R1 vs. R0			1.49 (1.28–1.72)^***^	1.21 (1.05–1.39)^***^
Nodal stage	N+ vs. N0			1.29 (1.04–1.60)^*^	1.49 (1.21–1.82)^**^

^*^ *p* ≤ 0.05, ^**^ *p* ≤ 0.001, ^***^ *p* ≤ 0.0001.

## DISCUSSION

The results of the present study identify nuclear ELAC2 expression as a weak to moderate prognostic feature in prostate cancers. Its prognostic impact is pronounced, however, in cancers lacking *TMPRSS2:ERG* fusion. The IHC analysis showed nuclear ELAC2 staining in 60.8% of the prostate cancers. The higher level of nuclear ELAC2 staining in cancers (35% with moderate to strong staining) as compared to normal prostate epithelial tissue (typically negative or only weakly positive) suggests that nuclear ELAC2 becomes up-regulated during malignant transformation in a subset of prostate cancers. Only one published study has evaluated ELAC2 protein expression by immunohistochemistry in prostate cancer [12]. These authors used a home-made antibody and described positive staining in basal cells of normal prostate and benign prostate hyperplasia (BPH) as well as tumor cell staining in all tested adenocarcinomas [12]. In line with our findings, tumor cell staining with prominent nuclear localization is also shown in “The human protein atlas” for the anti-ELAC2 antibody HPA019535 [22]. It is a limitation of our study that only one single 0.6 mm tissue spot per patient was analyzed, making it possible that the fraction of ELAC2 positive cancers was underestimated in case of tumor heterogeneity.

Nuclear ELAC2 overexpression is associated with adverse tumor phenotype in our study (advanced pT stage, high Gleason grade, lymph node metastases, and early biochemical recurrence, p < 0.0001 each). The reasons for tumor associated ELAC2 up-regulation are not known. Earlier work suggested that loss of ELAC2 might drive prostate cancer aggressiveness [23, 24]. Moreover, loss of ELAC2 function fits better to the concept of HPC2 as a mutated cancer susceptibility gene than its overexpression [5]. However, available data suggest multiple possible functions of ELAC2, one of which is potentially related to a cell growth pathway. ELAC2 knock down in prostate cells was shown to impact TGF-β/Smad signaling- mediated growth arrest [6]. The strong association seen with Ki67LI in this study supports an *in-vivo* role of ELAC2 protein in cell proliferation control. Of note, finding frequent overexpression of ELAC2 in our cancers does not exclude a tumor suppressive function. For example, we have previously analyzed the p16 tumor suppressor on the same TMA used in this study and made the paradoxical observation that p16 overexpression – and not loss - was linked to adverse tumor phenotype and poor prognosis [25]. Possible explanations include that tumor suppressors such as p16, and potentially ELAC2 as well, become up-regulated during tumor progression in an attempt to regain cell cycle control in response to deregulated growth signaling by other causes.

The highly annotated TMA allows us to draw some further conclusions on molecular mechanisms associated with ELAC2 up-regulation. About 50% of prostate cancers carry the gene fusion linking the androgen-regulated serine protease TMPRSS2 with the ETS-transcription factor *ERG,* resulting in the overexpression of ERG [17, 26, 27]. It has been shown that activation of the TGF-ß signaling pathway is one important consequence of ERG fusion in prostate cancer [27, 28]. The observed up-regulation of nuclear ELAC2 expression in the subset of ERG positive cancers might be caused by a general up-regulation of the TGF-ß pathway as a consequence of *ERG* fusion. Chromosomal deletions are another hallmark of prostate cancers. For our study, we selected deletions that others and us found to be associated with ERG-fusion positive (i.e. PTEN, 3p) [20, 21] or ERG-fusion negative cancers (i.e. 5q, 6q) [19]. That fractions of ELAC2 positive cancers were somewhat higher in PTEN and 3p deleted cancers likely reflects this association. However, a functional relationship between ELAC2 and the TGF-ß pathway cannot be excluded, as *PTEN*/AKT has been shown to modulate TGF-ß signaling through a direct interaction with Smad3 [29].

ERG activation modulates the expression of more than 1,600 genes, resulting in massive changes of the molecular environment of effected tumor cells [26, 27, 30, 31]. We identified various proteins with higher expression levels in the ERG positive than in the ERG negative subset. In some of these, the prognostic impact was reduced in the ERG positive subset and remained in the ERG negative subset [32–34]. Nuclear ELAC2 expression belongs to this group of proteins. Other biomarkers were only prognostic in ERG positive cancers [35, 36]. Overall, the data suggest that tumor relevant functions of ELAC2 and other proteins might be modulated by the ERG fusion status.

The Gleason score at prostatectomy is the strongest established prognostic parameter in prostate cancer. The ELAC2 analysis of tumors with matching Gleason grade largely demonstrates the power of morphology in the assessment of prostate cancer aggressiveness. With the exception of Gleason 3+4=7, the prognostic impact of ELAC2 expression was lost within traditional Gleason grade groups (Figure 6A). That Gleason 3+4=7 was the only group for which ELAC2 expression showed prognostic impact emphasizes that this group is the one with the most heterogeneous outcomes [37]. Many experts currently discuss the option of treating a fraction of Gleason 3+4 patients more conservatively with active surveillance [38]. By further refining the Gleason grading using the percentage of Gleason grade 4 as a continuous variable (quantitative Gleason Grade) [15], the prognostic impact of ELAC2 was even more reduced (Figure 6B–6H) to a small subset of patients with Gleason 3+4 cancers with a low fraction (≥30%) of Gleason 4 patterns. The case of ELAC2 demonstrates how the prognostic power of a molecular marker can depend on the quality of the histo-pathological diagnosis. However, it is a weakness of Gleason grading that inter-observer variability between pathologists generally exceeds 30% [39, 40]. We, thus, do not consider it as a disadvantage that the original Gleason grade from the patient’s files was used for statistical analyses. In addition, from 2005 on, Gleason grading was performed almost exactly to the WHO 2016 recommendation in our department. We therefore anticipate that ELAC2 analysis may aid in clinical decision making in situations where Gleason grading is particularly unreliable (such as small biopsies) and in cancers with small amounts of Gleason 4 patterns, most likely in concert with additional molecular markers.

In summary, nuclear ELAC2 overexpression is a frequent feature in prostate cancer with a potential role for tumor development and progression. The link between ELAC2 up-regulation and prostate cancer phenotype suggests a possible functional role of ELAC2 for the biology of the disease.

## MATERIALS AND METHODS

### Patients

The 12,427 patients had radical prostatectomy (RPE) between 1992 and 2012 at the University Medical Center Hamburg-Eppendorf (Department of Urology and the Martini Clinics). Specimens were analyzed with a highly standardized procedure [14]. Gleason grading was performed already from 2005 on as outlined by the WHO later in 2016 with minor modifications, i.e., we have a conservative position to define irregular glands as Gleason 4. The classical Gleason categories were supplemented with “quantitative” Gleason grading incorperating the percentage of Gleason 4 pattern [15]. Follow-up was available for 11,665 patients (median 50 months, range: 1 to 241 months; Table 6). PSA recurrence was defined as a PSA-level of ≥0.2 ng/ml and increasing after RPE. The TMA was manufactured from tumor blocks that were selected for a sufficiently high tumor cell content. One tumor block per patient was selected, and a single 0.6 mm punch was taken from each tumor block to manufacture the TMA as previously described with minor modifications [16]. The datafile attached to the TMA had results from earlier analysis of ERG expression [17], *ERG* break apart FISH analysis [18] and deletion status of 5q21 (*CHD1*) [19], 6q15 (*MAP3K7*) [19], 10q23 (*PTEN*) [20] and 3p13 (*FOXP1*) [21]. The ethics committee of the Ärztekammer Hamburg (WF-049/09) approved the study. Patient identification was anonymized such that, in accordance with local law (HmbKHG, §12a), no informed consent was required.

**Table 6:** Composition of the prognosis tissue microarray containing 12 427 prostate cancer specimens

	No. of patients	
	Study cohort on tissue microarray	Biochemical relapse among categories
**Follow-up**		
*n*	11 665	2 769 (23.7%)
Mean/Median (month)	62.9/50.0	
**Age (y)**		
≤50	334	81 (24.3%)
51–59	3 061	705 (23%)
60-69	7 188	1 610 (22.4%)
≥70	1 761	370 (21%)
**Pretreatment PSA (ng/ml)**	
<4	1 585	242 (15.3%)
4–10	7 480	1 355 (18.1%)
10–20	2 412	737 (30.6%)
>20	812	397 (48.9%)
**pT stage (AJCC 2002)**		
pT2	8 187	1 095 (13.4%)
pT3a	2,660	817 (30.7%)
pT3b	1 465	796 (54.3%)
pT4	63	51 (81%)
**Gleason grade**		
≤3+3	2 848	234 (8.2%)
3+4	6 679	1 240 (18.6%)
3+4 Tertiary 5	433	115 (26.6%)
4+3	1 210	576 (47.6%)
4+3 Tertiary 5	646	317 (49.1%)
≥4+4	596	348 (58.4%)
**pN stage**		
pN0	6 970	1 636 (23.5%)
pN+	693	393 (56.7%)
**Surgical margin**		
Negative	9 990	1 848 (18.5%)
Positive	2 211	853 (38.6%)

In the column “Study cohort on tissue microarray” numbers do not always add up to 12 427 in different categories because of cases with missing data. Percent in column “Biochemical relapse among categories” refers to the fraction of samples with biochemical relapse within each parameter in the different categories. Abbreviation: AJCC, American Joint Committee on Cancer.

### Immunohistochemistry (IHC)

Newly cut TMA sections were all stained in a single run. Slides were dewaxed, exposed to antigen retrieval (5 minutes at 121° C, pH 7.8 Tris-EDTA-citrate buffer), and incubated with primary ELAC2-specific antibody (rabbit polyclonal antibody, Novus Biologicals, Cambridge, NBP1-84620; dilution 1:50) at 37° C for 60 minutes. Binding was visualized with the EnVision Kit (Dako, Glostrup, Denmark). ELAC2 positive tissues showed uniform staining of all (100%) cell nuclei in the tissue spot. Thus, only the ELAC2 nuclear staining intensity was semiquantitatively assessed. A trained pathologist analyzed all slides by visual inspection at 100–200× maginification and estimated the staining intensity in four categories: negative, weak, moderate and strong (Figure 1).

### Statistics

Contingency tables and likelihood-tests were done to search for associations between molecular parameters and tumor phenotype. Kaplan-Meier survival curves were tested with the log-rank test. Cox proportional hazards were calculated to look for independent prognostic effects of various parameters in 4 clinical scenarios. All calculations were done with JMP 9 (SAS Institute Inc., NC, USA).

## SUPPLEMENTARY MATERIALS



## Abbreviations

cT: clinical stage
Li: labeling index
PSA: prostate specific antigen
pT: pathological stage
pN: nodal stage
R: surgical margin
TMA: tissue microarray
