# Supplementary material for: Nuclear ELAC2 overexpression is associated with increased hazard for relapse after radical prostatectomy
Source: Oncotarget. 2019 Aug 13;10(48):4973–86. doi: 10.18632/oncotarget.27132 (PMC6697635; doi:10.18632/oncotarget.27132)
Supplement: Supplementary file 1 [file oncotarget-10-4973-s001.pdf]

Nuclear ELAC2 overexpression is associated with increased hazard for relapse after radical prostatectomy

SUPPLEMENTARY MATERIALS

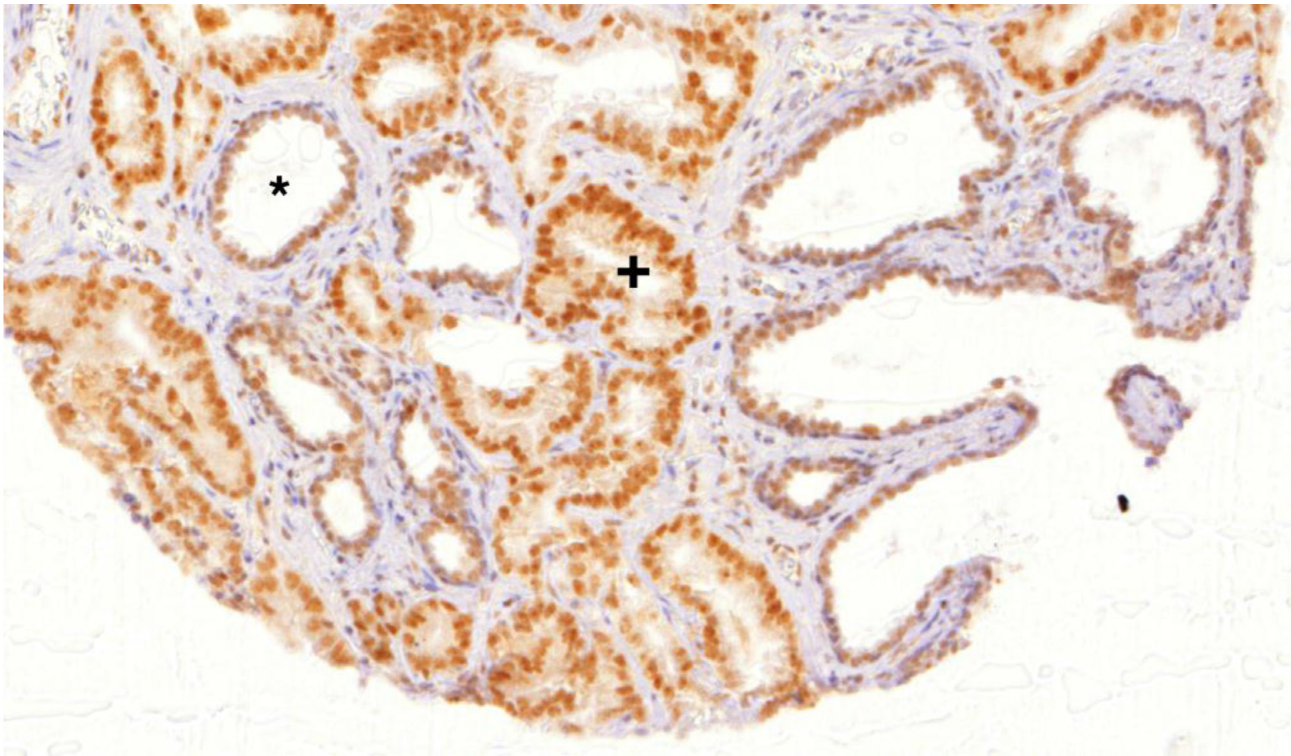

Supplementary Figure 1: Representative mixed spot with normal (*) and cancerous (+) glands at 400× original stained with anti-ELAC2 antibody NBP1-84620 at dilution 1:50.
